# Favorable QTLs from *Oryza longistaminata* improve rice drought resistance

**DOI:** 10.1186/s12870-022-03516-w

**Published:** 2022-03-23

**Authors:** Shaoying Huang, Manman Liu, Gaili Chen, Fengfeng Si, Fengfeng Fan, Yu Guo, Lei Yuan, Fang Yang, Shaoqing Li

**Affiliations:** State Key Laboratory of Hybrid Rice, Hongshan Laboratory of Hubei Province, Key Laboratory for Research and Utilization of Heterosis in Indica Rice of Ministry of Agriculture, Engineering Research Center for Plant Biotechnology and Germplasm Utilization of Ministry of Education, College of Life Science, Wuhan University, Wuhan, China

**Keywords:** Drought stress, QTLs, *Oryza longistamimata*, PEG6000, Principle component analysis

## Abstract

**Background:**

Drought is the major abiotic stress to rice grain production under unpredictable changing climatic environments. Wild rice of *O. longistaminata* show diverse responses and strong tolerance to stress environments. In order to identify whether the *O. longistaminata* can improve the rice drought resistance or not, a BIL population of 143 BC_2_F_20_ lines derived from the cross between the cultivar rice 9311 and *O. longistaminata* were assessed under stress of 20% PEG6000.

**Results:**

In total, 28 QTLs related to drought resistance based on eight agronomic traits of seedlings were identified. Of which, thirteen QTLs including two QTLs for leaf drying, one QTL for leaf rolling, one QTL for leaf number, five QTLs for dry weight of root, two QTLs for dry weight of shoot, one QTL for maximum root length and two QTLs for maximum shoot length were derived from *O. longistaminata*. What’s more, *qDWR8.1* for dry weight of root was repeatedly detected and fine-mapped to an interval about 36.2 Kb. The unique allele of MH08g0242800 annotated as ATP-dependent Clp protease proteolytic subunit from *O. longistaminata* was suggested as the candidate gene for drought resistance. Further, six representative BIL lines were stably characterized showing significantly stronger drought resistance than 9311 based on principle component analysis, they each contained 2 ~ 5 QTLs including *qDWR8.1* from *O. longistaminata*.

**Conclusions:**

Together, our results indicate that the QTLs from *O. longistaminata* can effectively enhance the drought tolerance of rice, showing great potential value in breeding of elite rice varieties, which will lay a novel insight into the genetic network for drought tolerance of rice.

**Supplementary Information:**

The online version contains supplementary material available at 10.1186/s12870-022-03516-w.

## Background

Rice (*Oryza sativa* L*.*) is the most economically important crop, and a staple food for more than half of the global population [1]. Rice yield is frequently limited by several abiotic factors such as cold, flood, and drought caused by climate change [2]. Climate related diverse abiotic stresses are the principal sources of risk and uncertainties in agriculture and caused wide fluctuations in agricultural output [3]. In recent years, drought stress has been witnessed in many parts of the world, breeding drought tolerance rice has become a priority research project [4], although numerous efforts to produce drought tolerant rice have been done around the world [5].

The genetics of drought resistance is quite complex in nature and associated with several quantitative traits. Plant roots are important organs to get water and nutrients [6]. Early and rapid root growth and elongation, seedling development are all important indicators of drought tolerance [7]. Improvement of drought tolerance of rice seedling can overcome the influence of water, establish advantage of the photosynthesis, and provide a solid prerequisite to obtain high and stable yield [8]. Therefore, seedling growth traits and their response to drought can be useful for the evaluation of drought-tolerant rice varieties [9]. The genus *Oryza* consists of cultivated species, *O. sativa* and *O. glaberrima*, and 22 wild species including *Oryza longistaminata.* Wild rice species are known to possess many valuable genes that do not exist in cultivated rice varieties [10]. However, only a few works regarding the cloning and QTLs mapping of valuable genes from wild rice species have been reported, systematically evaluation of wild rice and its derivative populations should be a priority in rice breeding programs.

*Oryza longistaminata*, an Africa wild rice, which is suggested as the ancient ancestor of cultivated rice, has numerous interesting traits, such as long stigma and large anthers, and high resistance to abiotic and biotic stresses including drought tolerance and bacterial blight resistance [10]. Precise and effective exploitation of the hidden valuable genes within *O. longistaminata* will help to ensure sustainable rice production and global food security in the ever-changing climatic conditions. Therefore, to illustrate *O. longistaminata*’s potential for improving drought-related traits, 143 BC_2_F_20_ lines derived from cultivated line 9311 and *O. longistaminata* were employed and analyzed by quantitative trait locus (QTL) under drought stress induced by 20% PEG6000, and 28 QTLs related to seven drought-related traits were identified. The genetic dissection of loci underlying drought stress in *O. longistaminata* will aid in our understanding of the genetics of drought tolerance, and will accelerate the development of new varieties with stronger drought tolerance.

## Results

### Observation on the phenotype the BILs under drought stress

The BIL population and 9311 were evaluated under artificial drought stress and normal growing conditions in greenhouse, and the differences and variations of phenotype of BILs were summarized in Table 1 and Table S2. Under 20% PEG6000 stress, the leaf number, dry weight of shoot and shoot length of BILs were inhibited to some extent (Table 1). However, the dry weight of root was significantly increased compared to the CK group, indicating that increasing biomass of root will help rice to resist the drought stress. Leaf rolling and leaf drying are two critical and useful traits for intuitive judgment of the drought tolerance of BILs. Delaying in leaf drying and leaf rolling of a few of BILs with lower scores helped them to improve drought tolerance. Across the populations, the score of leaf rolling and leaf drying showed a wide variation from 1.67 to 9.0 and 1.0 to 8.33, respectively under drought stress in the both experiments (Table 1).

**Table 1:** Phenotype performance of BILs under artificial drought stress

Experiment	Traits	Treatment	9311	BILs				
				Mean	Max	Min	SD	CV (%)
Exp 1	LD	T	4.33	4.04	8.33	1.00	1.34	33.1
	LR	T	5.00	5.14	9.00	1.67	1.83	35.6
	LN	CK	5.00	4.98	6.00	4.00	0.35	7.1
		T	4.00	4.04	5.00	3.00	0.31	7.6
	DWR (mg)	CK	11.67	11.66	23.33	5.00	4.37	37.4
		T	15.44	16.41	30.22	8.33	4.47	27.2
	DWS (mg)	CK	47.89	54.21	112.89	18.89	21.30	39.3
		T	49.00	46.51	91.44	19.67	15.85	34.1
	RL (cm)	CK	14.98	11.80	20.34	7.24	2.09	17.7
		T	12.82	10.97	14.51	6.18	1.42	12.9
	SL (cm)	CK	32.37	38.37	59.33	24.53	8.86	23.1
		T	25.54	28.99	47.11	17.99	7.59	26.2
	RS ratio	CK	0.46	0.32	0.46	0.17	0.06	17.8
		T	0.50	0.40	0.64	0.23	0.09	22.9
Exp 2	LD	T	5.67	4.76	8.33	1.00	1.36	28.5
	LR	T	5.00	5.33	9.00	1.67	1.56	29.2
	LN	CK	5.00	4.71	6.00	4.00	0.38	8.0
		T	4.00	3.96	4.67	3.00	0.23	5.9
	DWR (mg)	CK	11.22	8.99	19.33	3.89	3.54	39.3
		T	15.11	12.37	27.11	6.11	3.67	29.6
	DWS (mg)	CK	47.56	38.06	82.67	12.78	14.85	39.0
		T	45.44	35.17	81.44	15.56	11.89	33.81
	RL (cm)	CK	12.57	11.53	15.59	6.42	1.64	14.2
		T	14.57	13.03	17.68	9.27	1.66	12.7
	SL (cm)	CK	33.44	34.50	56.42	21.58	8.84	25.6
		T	26.77	26.48	43.47	17.78	6.20	23.4
	RS ratio	CK	0.38	0.35	0.56	0.17	0.09	25.5
		T	0.54	0.51	0.82	0.31	0.11	20.8

*Notes*: *LD* represents leaf drying, *LR* represents leaf rolling, *LN* represents leaf number, *DWR* represents dry weight of root, *DWS* represents dry weight of shoot, *RL* represents maximum of root length, *SL* represents maximum of shoot length, *RS* ratio represents value between maximum root length and shoot length. *CV* represents the coefficient of variation. *SD* represents standard error. *Max* represents maximum. *Min* represents minimum. *Exp* represents experiment

**Fig. 1:**
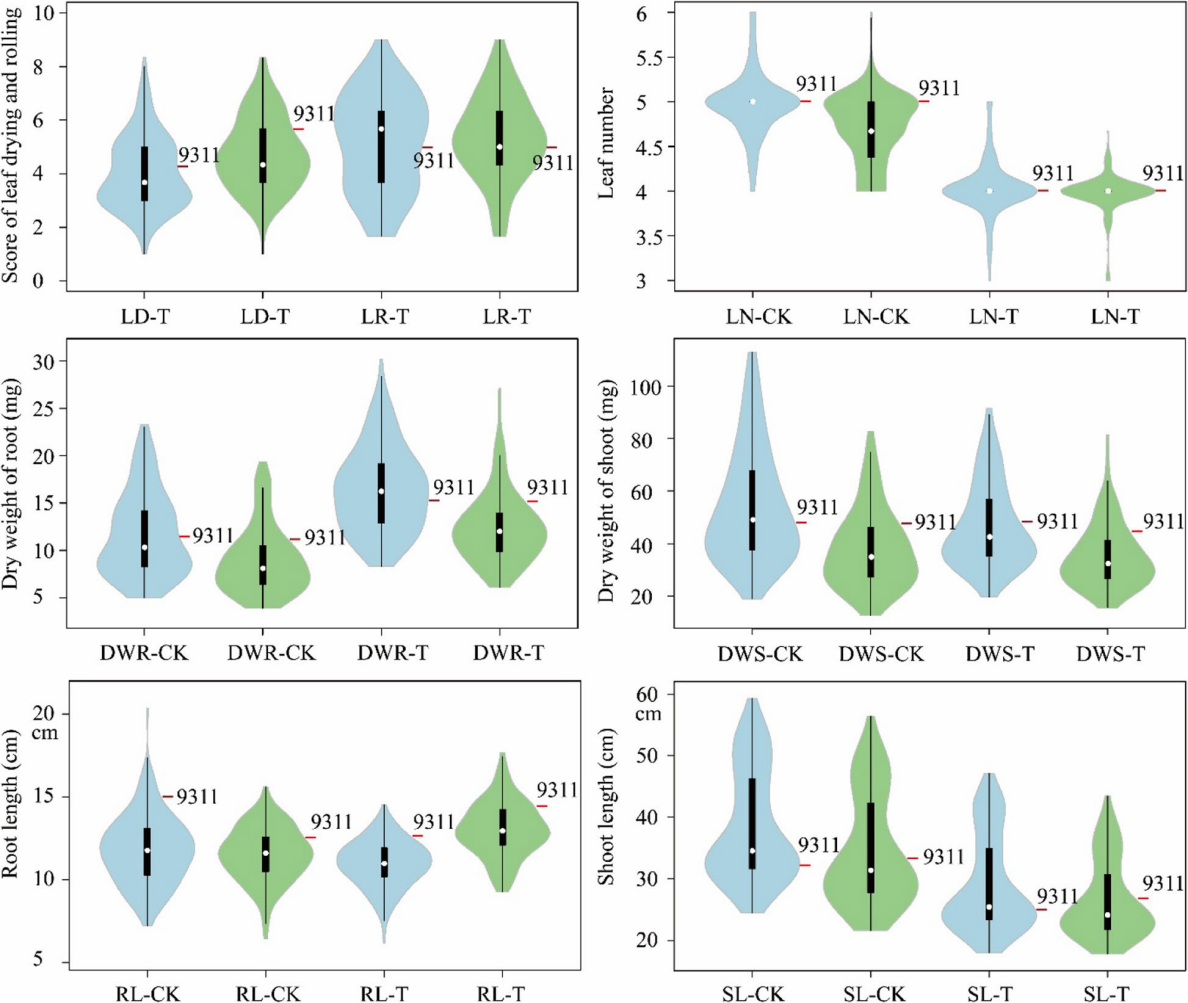
Distribution of the seven drought-resistance related traits of *O. longistaminata* BILs. light blue represents Exp.1, and light green represents Exp.2. Red line represents the value of 9311

When compared the distribution pattern of the agronomic traits of the *O. longistaminata* BIL population, it is apparently found that each of the traits showed a similar distribution pattern between CK and treatment, except the leaf number in the second experiment (Fig. 1), reflecting the stability and consistency of the experiments. Most of the traits in BIL population showed a normal, or approximately normal distribution patterns, except LD-T, LR-T, DWRCK, SLT and SLCK in Exp.1, and LNCK, SLT and SLCK in Exp.2 (Fig. 1). Deviation of SL distribution in BILs could be the result of the influence of some major genes and modifiers. Compared to normal conditions, the variation’s average of DWR in Exp.1 and Exp.2 had obvious increase in the whole population, which is opposite to the other traits, reflecting the special response of DWR different from the other traits to the drought stress.

 Damage rate of the traits showed that, relative to the parent 9311, there were 55.2% and 69.9% of BIL lines showing less leaf drying, and 39.9% and 31.5% BIL lines showing leaf rolling in the two experiments (Table S2). For the other traits, there were about 34.3% and 53.9% lines for leaf number, 28.7% and 34.3% lines for shoot length, 71.0% and 39.9% lines for root length, 26.6% and 46.6% lines for dry weight of shoot, 30.8% and 42.7% lines for dry weight of root showed less damage in the two experiments than the 9311 (Table S2), respectively, which indicate that these BIL lines have stronger resistance to the drought tolerance than 9311 in the respective specific traits. Correspondingly, in the first experiment, there were thirteen lines showed at least five traits superior to the 9311 under drought stress. In the second experiment, there were twenty lines showed at least five traits superior to the 9311 under drought stress (Table S3).

### Correlation analysis of the drought related traits

Coefficients of variation (CV) can well reveal the genetic and phenotypic divergence and sensitivity of a population to an adverse factor to some extent, and is widely used for measure of dispersion. Analysis showed that the coefficients of variation of the investigated traits had great phenotypic variability in the BILs, the coefficients of variation of the eight traits ranged 7.6/5.9 to 35.6/29.2 in drought stress, and the six traits ranged 7.1/8.0 to 39.3/39.0 in normal condition (Table 1). The leaf number (LN) was recorded the lowest CV (< 10%), and the dry weight of root (DWR) was recorded the highest CV (> 30%) in the experiments. The other six traits including root length (RL), shoot length (SL), dry weight of shoot (DWS), leaf drying (LD), leaf rolling (LR), and ratio of root length/shoot length (RS ratio) were recorded medium CV (Table 1). Which mean that the dry weight of root is the most sensitive agronomic trait to the stress of PEG6000 in the eight investigated traits of seedlings, and may be a critical parameter to assess the tolerance of rice to the drought stress.

**Fig. 2:**
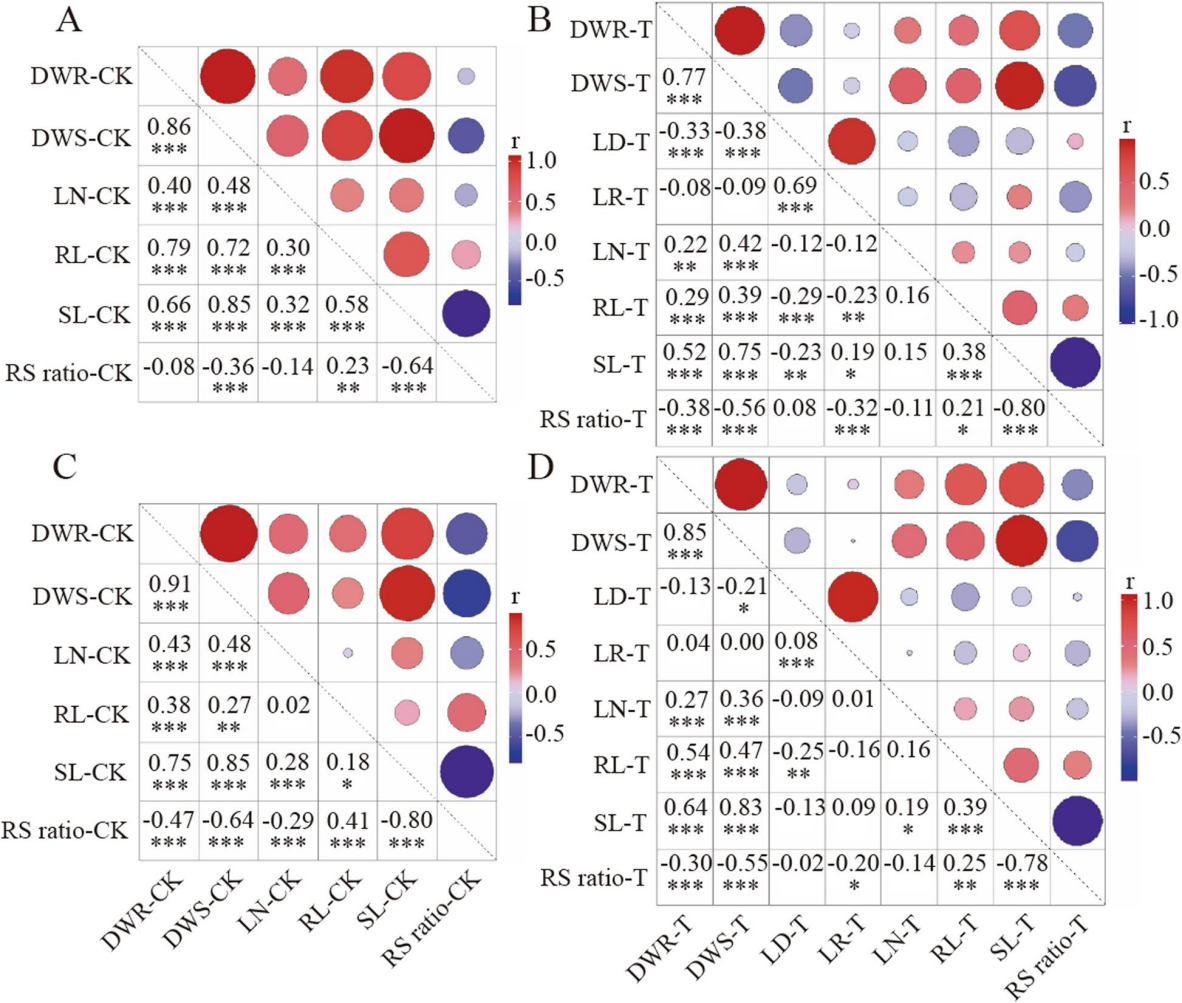
Graphical correlation matrix for drought-related traits of BILs. **A** correlation coefficients of CK in Exp.1. **B** correlation coefficients of treatment in Exp.1. **C** correlation coefficients of CK in Exp.2. **D** correlation coefficients of treatment in Exp.2. *, ** and *** mean significance at *P* < 0.05, 0.01 and 0.001, respectively. r means correlation coefficient

Correlation analyses of the eight traits in the treatment and the six traits in the CK group showed that, in CK group, except no difference of RS ratio to DWR or LN in Exp.1 and RL to LN in Exp.2, all other traits had significant or extremely significant positive or negative correlations each other. The DWR showed extremely significant positive correlation with SL, RL, DWS and LN, while RS ratio showed extremely significant negative correlation with others. When treated with PEG6000, the DWR also showed a similar positive correlation with SL, RL, LN and DWS as that in normal growth, and the RS ratio showed extremely significant negative correlations with the other traits (Fig. 2). It indicates that the performance of root had relatively significant influence on the other traits of plants. As all measured root-related traits are associated in positive direction, they could simultaneously improve drought tolerance. The LD and LR showed an extremely significant positive correlation in the two experiments (*r* = 0.69, 0.80, respectively). The LD showed significant or extremely significant negative correlation with RL or DWS in the two experiments, and extremely significant negative correlation with SL or DWR in the Exp.1. The LR showed extremely significant negative correlation with RS ratio or RL in the Exp.1, and significantly positive correlation with SL in the Exp.1, meaning that the leaf drying and leaf rolling will greatly prevent the growth of shoot and root of rice when treated with PEG6000.

### Principal component analysis and comprehensive evaluation of phenotypic traits

**Fig. 3:**
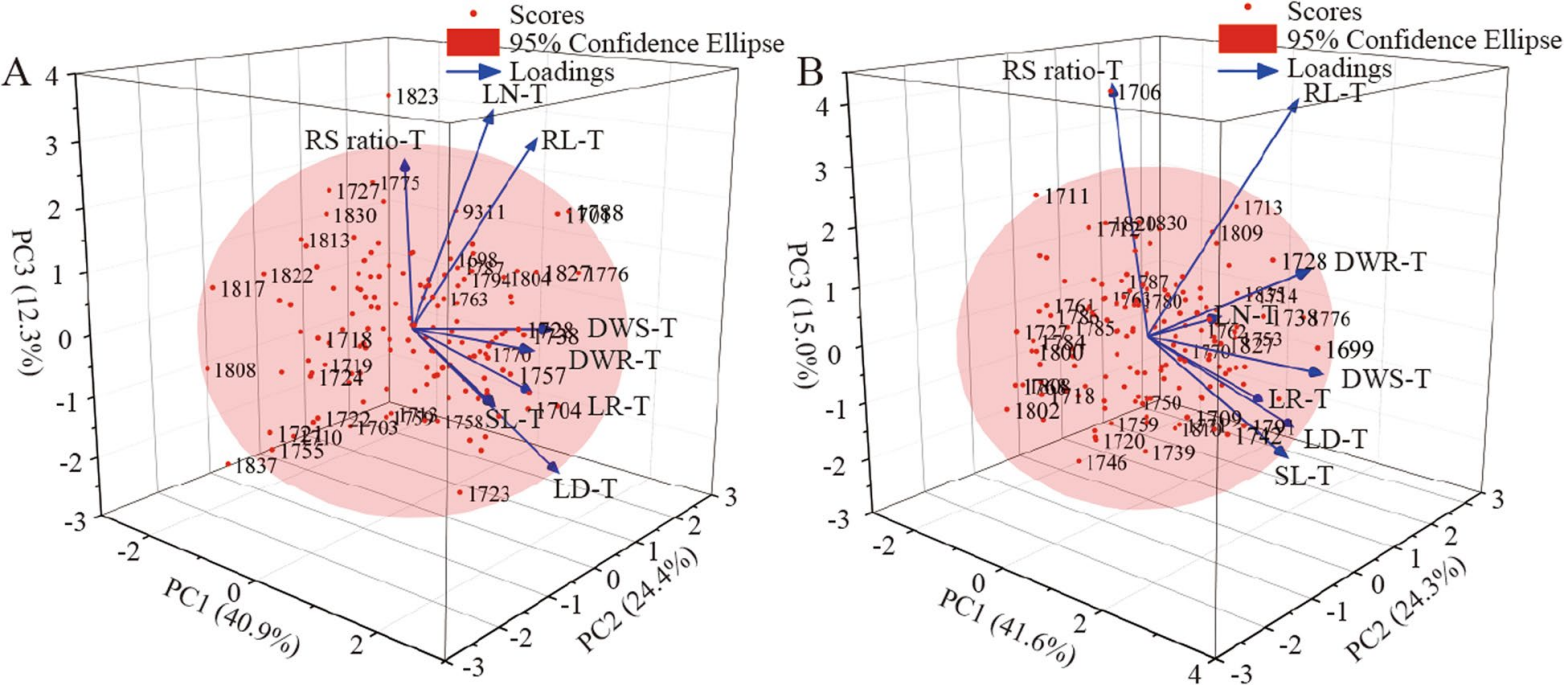
3D scatter plot of the tested BIL lines based on the first, second and third principle component in the Exp.1 (**A**) and Exp.2 (**B**). T means treatment, LD means leaf drying, LR means leaf rolling, LN means leaf number, DWR means dry weight of root, DWS means dry weight of shoot, RL means maximum of root length, SL means maximum of shoot length, RS ratio means value between maximum root length and shoot length. The confidence ellipse represents the 95% confidence interval for different species

In order to discover the critic genetic parameters for drought resistance and elite BIL lines against drought stress, the BIL lines were comprehensively evaluated by using subordinate function values analysis combined with the principle component analysis (Table 2, Table S4, Fig. 3), then the three PCs close to or greater than 1 of eigenvalue were extracted for synthetical evaluation (F value) in the two experiments (Table S5). The eigenvalues of PC1, PC2 and PC3 were greater than one in the two experiments, except the eigenvalue of PC3 in Exp.1 was 0.98. The contribution rate (CR) of PC1 in the two tests were more than 40%, the traits of DWS, SL and DWR with positive load contributed most of the PC1 in the two tests, indicating that these traits are more important to improve the drought resistance. The contribution rate (CR) of PC2 in Exp.1 and Exp.2 were 24.43 and 24.03%, the LD, LR and RS ratio with positive load contributed most of the PC2. The contribution rate of PC3 in the first experiment were 12.3%, the LN, RL and RS ratio of traits with positive load contributed most to it. The contribution rate of PC3 in Exp.2 were 15%, the RL and RS ratio with positive load contributed most to it. The cumulative contribution rate of PC1, PC2 and PC3 were close to 80% in the first experiment, and greater than 80% in the second experiment. Further, the PCA analysis combined with the synthetical evaluation found that the BIL 1776 had a best performance, because the 1776 was ranked at the third and fourth in the first and second experiment, respectively, prominently superiorior to the 9311 which was ranked at the 35th in the Exp.1 and 33th in the Exp.2. While, the 1808 ranked at the last one in the two experiments. Thus, the BIL line 1776 and 1808 were suggested as two representative lines in the BIL population according to the trait of DWS, SL, DWR, LR, LD and RS ratio.

**Table 2:** Power vector (PV) of selected principal components for eight traits related to drought resistance among BILs

Trait	Exp	PV1	PV2	PV3	Exp	PV1	PV2	PV3
LD	1	0.27	0.47	-0.45	2	0.14	0.61	-0.34
LR	1	0.07	0.63	-0.29	2	0.01	0.64	-0.30
LN	1	0.22	0.11	0.58	2	0.22	0.02	0.09
DWR	1	0.44	0.02	0.01	2	0.47	0.02	0.25
DWS	1	0.52	-0.02	0.09	2	0.53	-0.02	0.02
RL	1	0.25	0.32	0.49	2	0.28	0.29	0.60
SL	1	0.47	-0.25	-0.07	2	0.50	-0.14	-0.16
RS ratio	1	-0.35	0.45	0.36	2	-0.33	0.34	0.58

*Notes*: *Exp*. Experiment, *PV* Power vector

### QTL mapping for drought tolerance

**Fig. 4:**
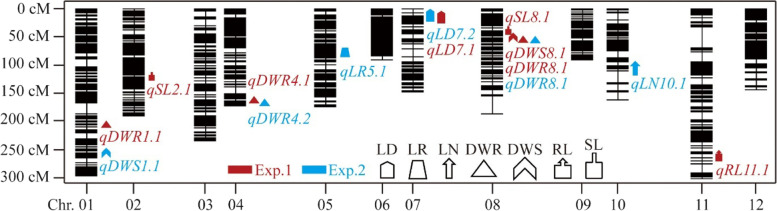
QTLs for drought resistance from *O. longistaminata* with additive effect. LD, leaf drying; LR, leaf rolling; LN, leaf number; DWR, dry weight of root; DWS, dry weight of shoot; RL, maximum root length; SL, maximum shoot length; RS ratio, between maximum root length and shoot length. Red means Exp.1, Blue means Exp.2

Using the high-density SNP linkage map and phenotypic data of seedling traits under drought conditions, a total of 28 QTLs were detected in BILs populations for eight traits. The QTLs were located on all chromosomes, except chromosome 3 and 12 (Fig. 4, Table 3, Table S6).

**Table 3:** QTLs with additive effect for drought resistance identified from *O. longistaminata*

Trait	QTLs	Exp	Chr	Position	L-Marker	R-Marker	LOD	PVE (%)	Add
LD	*qLD7.1*	1	7	41.5 cM	BIN7-6	BIN7-7	3.06	9.93	0.95
	*qLD7.2*	2	7	29.5 cM	BIN7-3	BIN7-4	4.39	10.84	1.04
LR	*qLR5.1*	2	5	134.97 cM	BIN5-113	BIN5-114	3.85	10.83	0.97
LN	*qLN10.1*	2	10	168.9 cM	BIN10-107	BIN10-108	11.13	10.70	0.33
DWR	*qDWR1.1*	1	1	217.45 cM	BIN1-161	BIN1-162	9.57	16.36	2.10
	*qDWR4.1*	1	4	229.53 cM	BIN4-161	BIN4-162	7.94	13.50	2.00
	*qDWR4.2*	2	4	246.53 cM	BIN4-196	BIN4-197	3.97	0.68	1.15
	*qDWR8.1*	1	8	76.58 cM	BIN8-58	BIN8-59	4.58	8.22	2.66
	*qDWR8.1*	2	8	76.58 cM	BIN8-58	BIN8-59	4.95	1.08	2.35
DWS	*qDWS1.1*	2	1	266.45 cM	BIN1-234	BIN1-235	3.78	22.47	3.76
	*qDWS8.1*	1	8	72.58 cM	BIN8-58	BIN8-59	3.68	9.75	8.32
RL	*qRL11.1*	1	11	371.91 cM	BIN11-173	BIN11-174	3.92	9.48	1.39
SL	*qSL2.1*	1	2	178.43 cM	BIN2-232	BIN2-233	4.89	7.15	2.31
	*qSL8.1*	1	8	57.58 cM	BIN8-54	BIN8-55	3.33	4.77	1.97

*Notes*: *PVE* means percentage of phenotypic variance explained by each QTL, *LOD* means logarithm of odds. *Exp.1 and Exp.2* represent experiment 1 and experiment 2, *Chr*. means chromosome

In the first experiment, 13 QTLs were detected for seven different traits (LD, LN, DWR, DWS, RL, SL and RS ratio), which explained 4.77% to 43.6% of the phenotypic variations (Table 3, Table S6). Of which, eight QTLs with additive effects were contributed by *O. longistaminata* (Table 3), the *qLD7.1* for leaf drying with a LOD of 3.06 explained 9.93% of the phenotypic variation, the *qRL11.1* for root length explained 9.49% of the phenotypic variation, and the *qDWS8.1* for dry weight of shoot with a LOD of 3.68 explained 9.75% of the phenotypic variation. The *qSL2.1 and qSL8.1* for shoot length explained 7.15% and 4.77% of the phenotypic variations, respectively. The *qDWR1.1, qDWR4.1* and *qDWR8.1* for dry weight of root were identified with a PVE of 16.36%, 13.49% and 8.21% respectively, and the *qRSratio1.1* for maximum root length/maximum shoot length ratio explained 43.6% of the phenotypic variation (Table 3).

In the second experiment, a total of 16 QTLs were identified for seven different traits in BILs, which explained 0.5% to 42.13% of PVE (Table 3, Table S6). Of which, six QTLs were contributed from *O. longistaminata* (Table 3). The *qLD7.2* for leaf drying with a LOD of 4.39 on chromosome 7 explained 10.84% of the phenotypic variation, and the *qLR5.1* for leaf rolling with a LOD of 3.85 explained 10.83% of the phenotypic variation. The *qLN10.1* for leaf number with a LOD of 11.13 explained 10.70% of the phenotypic variation. The *qDWS1.1* for dry weight of shoot with a LOD of 3.78 explained 22.47% of the phenotypic variation, and the *qDWR4.2* and *qDWR8.1* for dry weight of root explained 0.68% and 1.08% of the phenotypic variations, respectively (Table 3). In all of the 28 QTLs, the *qDWR8.1* was repeatedly detected in the two tests.

Related traits are often due to pleiotropic effects of QTLs, which may enable selection for a complex trait via an easily observable trait. In the present study, four loci distributed over chromosomes 1, 7, 8 and 9 have been found to harbor multiple QTLs affecting the same or different traits of the BIL lines. The number of QTLs in each of the clusters ranges from 2 to 3. Within a region of 5 cM, three QTL were located on chromosome 1 for *qDWR1.1* (Table 3), *qRSratio1.1* and *qRSratio1.1* (Table S6). In Exp.2, QTL *qRL7.1* and *qDWR7.1* co-localized on chromosome 7 at peak of 110.5 cM that genomic region contributed by 9311 (Table S6). Similarly, the region of 2 cM on chromosome 9 has QTLs cluster (*qLN9.1 and qLR9.1*) related to leaf number and leaf rolling, which were also supplied alleles by 9311 (Table S6). Especially, in an interval of 4 cM on chromosome 8, two QTLs for *qDWS8.1* and *qDWR8.1* were clustered and showed the positive effect from the allele of *O. longistaminata* (Table 3). Notably, the DWR were associated with three of four clusters, so this trait can be used as an important indicator for drought tolerance.

### Confirmation and Candidate gene analysis of *qDWR8.1* for drought resistance

**Fig. 5:**
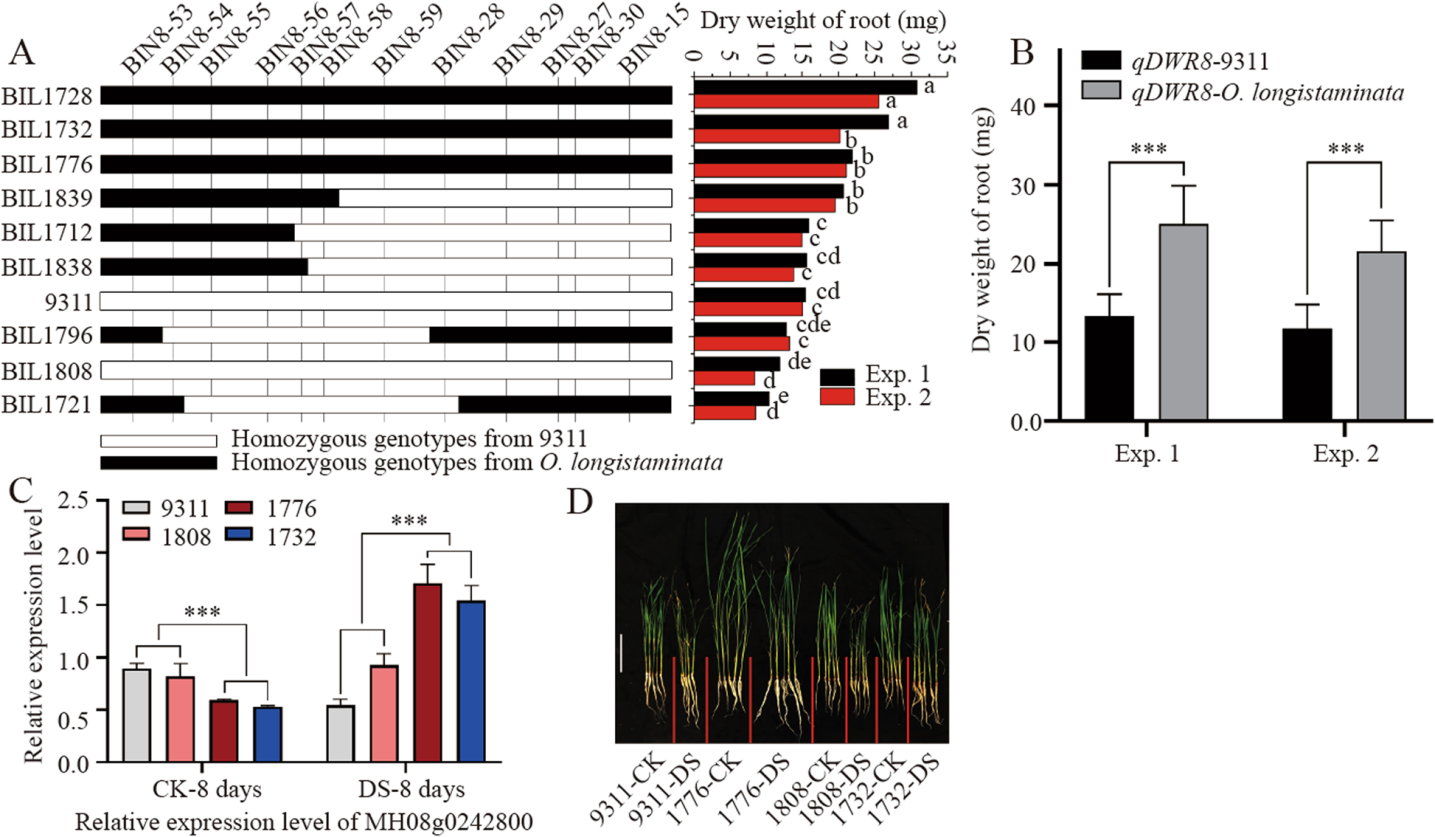
Functional confirmation and identification of candidate gene of qDWR8.1. **A** Genotype of the representative BIL lines at the locus of qDWR8.1. White and black indicate the fragment from 9311 and O. longistaminata, respectively. **B** Dry weight of root of the corresponding BIL lines in the two experiments. **C** Relative expression level of the candidate gene MH08g0242800 under drought stress. Lines 1776 and 1732 with qDWR8.1 contain alleles from *O. longistaminata*, 1808 contain alleles from 9311. **D** Images of 9311 and BILs in normal growth and drought stress (10 days). CK means normal growth, DS means drought stress with 20% PEG6000. *** mean significance at *P* < 0.001. Bar represents 10 cm

In order to confirm the genetic function of the newly identified drought-resistant locus from *O. longistaminata*, the *qDWR8.1*, which contribute most to the drought-resistance, were selected for further analysis*.* Based on the high-resolution Bin map of the BIL population, the *qDWR8.1* was delimited by the BIN8-58 and BIN8-59 to an interval about 36.2 Kb referenced to the MH63 genome. Genotypic analysis showed that the BIL lines 1728, 1732, 1776 and 1839 harboring the *qDWR8.1* showed consistently different structure from that of the BIL lines 1712, 1721, 1796, 1808, and 1838 without *qDWR8.1*. Correspondingly, the dry weight of root of the BIL lines 1728, 1732, 1776 and 1839 were all significantly larger than that of the BIL lines 1712, 1721, 1796, 1808, and 1838 and 9311 (Fig. 5A), and the average DWR of the lines with *qDWR8.1* was 25.06 in Exp.1 and 21.61 in Exp.2, which was significantly higher than those lines without *qDWR8.1*, (14.22 in Exp.1 and 12.61 in Exp.2, *P* < 0.001) (Fig. 5B), meaning the *qDWR8.1* from *O. longistaminata* could really improve the drought resistance of the BIL lines.

Further analysis showed that there were six ORFs in the locus of *qDWR8.1*, including three functional genes and three hypothetical genes (Table 4). Expression analysis of the representative BIL line seedlings showed that the expression of *MH08g0242800* in resistant BIL1776 and 1732 was significantly lower than that in sensitive BIL1808 and 9311 in normal growth condition. However, when treated with PEG6000, the expression of *MH08g0242800* was significantly induced in BILs 1776 and 1732 (Fig. 5C). The MH08g0242800 was suggested to encode an ATP-dependent Clp protease proteolytic subunit, which is involved in the process of shoot system development and cell viability in plants [11], and plays a major role in the degradation of misfolded proteins to induce the extracytoplasmic-stress response in *E. coli* and *S. typhimurium* [12, 13], meaning the function of MH08g0242800 is most possibly involved in resistance to abiotic stress. Bioinformatic analysis showed that the promoter of MH08g0242800 in *O. longistaminata* has two more “abscisic acid responsiveness” and “endosperm expression” cis-elements than that of 9311, and there were six SNPs, 5 bp insertion and 38 bp deletion of nucleotides in the coding region of MH08g0242800 in 9311. Of which, five SNPs lead to variation of amino acids, and the 5 bp insertion and 38 bp deletion of nucleotide in 9311 result in loss of a loop and a β-shift of the encoded protein (Figure S2), which may affect the biochemical function of MH08g0242800 encoded protein and drought resistance of rice. Moreover, the BIL 1776 and 1732 showed apparently higher surviving rate than that of the 9311 and BIL 1808 after treated with PEG6000, indicating the *qDWR8.1* help to increase the surviving rate of BIL lines (Fig. 5D).

**Table 4:** The predicted functional genes at locus of *qDWR8.1*

Locus names	CDS coordinates	Gene function
MH08g0242500	Chr08:9,978,993..9979181	Hypothetical protein
MH08g0242600	Chr08:9,986,888..9987971	Hypothetical protein OsI_28670
MH08g0242700	Chr08:9,991,878..9994124	Hypothetical protein OsI_28672
MH08g0242800	Chr08:10,002,492..10002806	ATP-dependent Clp protease proteolytic subunit
MH08g0242900	Chr08:10,004,451..10004828	30S ribosomal protein S18, chloroplastic
MH08g0243000	Chr08:10,008,081..10017754	Large proline-rich protein BAG6

*Notes*: *Cds* means coding sequence

### Genotypic analysis of the representative drought-resistant BIL lines

In order to further understand the genetic effects of QTLs from wild rice *O. longistaminata* on the improvement of rice drought-resistance, we compared the BIL lines superior to 9311 in drought resistance in the two experiments based on the principle component analysis. Results showed that there were 34 and 32 BIL lines superior to 9311 in the Exp. 1 and Exp. 2 (Table S5), respectively. Of which, there were six BIL lines of BIL 1702, 1704, 1728, 1732, 1742, 1776, showing stronger resistance in the two experiments, they harbored four, four, five, three, four and two QTLs from *O. longistaminata*, respectively (Supplementary Table S7). Especially, the BIL 1704, 1728 and 1776 were stably ranked in the top 10 of the BIL lines for strong drought-resistance in the two experiments, and all of them contained the *qDWR8.1/qDWS8.1*, consistent with their great additive effects for the phenotype (Table 3), meaning that this QTL cluster from *O. longistaminata* plays a critical role in enhancing rice drought resistance.

## Discussion and conclusion

Drought tolerance is a complex quantitative trait with a complicated phenotype that controlled by multiple genes with a relatively small effect [14]. Principle component analysis is a powerful tool for manage multiparametric data matrices and quantify genetic divergence of populations with respect to characters [15]. The PCA will increase the accessibility for the biologist and plant breeders for researching the number of plants to be assessed and characteristics that could be used to select the genotypes of drought tolerance [16]. As showed in Table S4, the cumulative contribution rate of first three PC was closely or greater to 80% in the two experiments. Of which, the cumulative contribution of first second PC was up to 65.32% and 65.89% in the two experiments, indicated the DWS, SL, DWR, LD, LR and RS were the most contributed traits to PC1 or PC2 in the two experiments under stress. In Fig. 3 and Table S5, the advantages of BILs in these traits were showed through the 3D plot analysis of PC1, PC2 and PC3 in the two experiments under drought stress. These results indicate that the DWS, SL, DWR, LD, LR and RS ratio can be used to prime discriminatory traits for drought resistance. Similarly, the rotated component matrix of nine phenotypic from 60 BILs plus two parents and four controls were evaluated by PCA under drought stress conditions. The degree of SL, RL, FRW and DWR was obviously higher than the other traits to PC1, level for LR to PC2 [17]. PCA on fourteen traits of 24 rice genotypes were evaluated under control and drought stress, which had produced consistent results [18]. There is a bright spot among these results that the DWR had greater contribution to PC1, indicated the trait can be used as an important indicator for seedling drought stress.

Phenotypic plasticity is one of the main strategies of plants to adapt to abiotic stresses via changes in critical developmental stages. Diversity for plasticity is commonly found in wild crops adapted to their environments [19]. In seasonally dry areas of Africa, *O. longisstaminata* accessions are often found showing great increase in total root mass [20, 21]. In our experiments, when the BIL lines were treated with PEG6000, the dry weight of root of BIL lines significantly increased compared with normal growth group in the two tests (Fig. 1), this is consistent with the principle component analysis (Table 2, Table S5), and the characters of cultivars and *O. longistaminata* to increase dry weight of root under dry or PEG stresses [22], meaning that the root is most sensitive to drought stress than the other organs, and maintaining a higher root biomass may help to enhance adaptability of BILs to drought stress.

At present, there are 653 QTLs for drought tolerance having been identified in rice based on 13 traits [23]. In our study, 13 QTLs related to drought resistance were detected from *O. longistaminata* with additive effect based on the seven seedling traits. Of which, the *qDWR8.1* was repeatedly detected in the two experiments and showed great genetic effects on the phenotypic variations (Table 3). Combined with the principle component analysis, the dry weight of root was the most stable and effective indicator to assay the drought resistance of rice. Importantly, the *qDWR8.1* detected in our study does not overlap with any other previously identified six QTLs related to DWR on chromosome 8 [24–26]. Moreover, the *qDWR8.1* for dry weight of root was delimited to an interval about 36.2 Kb with three functional genes by BIN8-58 and BIN8-59, and further bioinformatic analysis and qRT-PCR showed that only the MH08g0242800, which encode a ATP-dependent Clp protease proteolytic subunit, had not only great sequence variations in the promoters and gene body, but also significantly higher expression in BILs with *O. longistaminata* allele than 9311 and the lines harboring 9311 allele (Fig. 5C, Supplementary Figure S1), meaning it is most possibly the candidate for drought tolerance in *O. longistaminata*, although it is needed to be confirmed by further genetic complimentary test.

In this research, there were six BIL lines with 2 ~ 5 QTLs from *O. longistaminata* showing superior to 9311 in the two experiments according to PCA and comprehensive evaluation (Fig. 3, Table S7), indicating that the elite alleles from *O. longistaminata* can effectively improve the resistance of rice to drought stress. Correspondingly, there were 13 novel QTLs different from previous reports [23] identified from wild rice *O. longistaminata* (Fig. 4), which will provide an alternative for improving drought resistance of the rice varieties through molecular breeding, and settle an important step to figure out the molecular basis of drought tolerance.

## Methods

### Plant materials

A total of 143 BC_2_F_20_ BILs, developed from the cross between wild rice *O. longistaminata* and elite indica cultivar 9311 as recurrent parent in May 2021 in Lingshui autonomous region, Hainan province, China, was used for artificial drought stress with 20% PEG6000. All the plant seedlings were grown incubated in greenhouse at 25 °C ± 2 °C (day/night), with a 14-light/10-h dark photoperiod (irradiance: 400 μmol m^−2^ s^−1^) in Wuhan city, Hubei province, China. The experiments of drought treatment were repeated two times, and each time consisted of three technical replicates. 

### Artificial drought treatment

The rice seed treatment was dried at 42 °C for 24 h in constant drying oven, then soaked and germinated seed treatment in ddH_2_O for 36 h. Then, 96 uniform plumules of each line were selected and transplanted equally to six 96 well hydroponic box (16 plants per box) with ddH_2_O for 7 days, of which, three boxes as the control group and the other three boxes as drought stress group. The ddH_2_O was renewed every 56 h. After 7 days, the ddH_2_O was replaced by the nutrient solution, and the pH of nutrient solution was maintained at 5.0–5.8 with 1 M KOH during the entire growth period [27], and the nutrient solution renewed every 72 h. After 15 days growth, the treated groups were cultured in nutrient solution with 20% PEG6000 (w/v) for 10 days, and every 5 days the culture solution were re-changed with fresh solution with PEG [28]. The control groups were cultured with nutrient solution all the time.

### Investigation of phenotypic traits

After seedling washed three times with ddH_2_O, phenotypes of maximum root length (RL, the longest root), maximum shoot length (SL), maximum root length/maximum shoot length ratio (RS ratio), leaf rolling score (LR), leaf drying score (LD) and leaf number (LN) were investigated instantly, and the drought-stress damage parameter were scored based on leaf rolling and leaf drying according to the International Rice Research Institute (IRRI) standard after 10 days of treatment [29]. Then, the roots and shoots were cut off to dry at 80 °C to constant weight for dry weight of roots (DWR) and dry weight of shoot (DWS), respectively. The damage rate (Dam R) was calculated according to the following formula: Dam R = (CK – T)/CK × 100. Each trait of every line was measured three plants, excluded border plants to avoid edge effects, and repeated three times included control and treatment group.

### Data analysis

Phenotypic variance was calculated using Microsoft Excel software. The computation and visualization of Pearson’s correlation coefficient values among traits were used to obtain by RStudio version 1.4.1717 with the R package *Corrplot* [30]. The average of traits was transferred by subordinate function values analysis before principal component analysis (PCA) by Microsoft Excel software. The multiple comparison of traits with mean value and PCA of traits with subordinate function values were calculated by DPS 7.05 software. The 3D plot of PCA was generated with Origin 2018 software. The distribution of traits was drawn by an online tool [31].

### QTL analysis

The QTL IciMapping 4.1 software was used to construct the linkage map and QTL analyses [32]. The construction of genetic linkage map covering the whole genome was described by Jin et al. [33]. QTL mapping was carried out using the BIP functionality (QTL mapping in biparental populations). The inclusive composite interval mapping of additive (ICIM-ADD) QTL method with default option was used to detect additive QTLs. An odds (LOD) threshold value of 2.5 was applied. The putative genes in the QTL region were identified through the database of RIGW (Rice Information Gateway) [34].

### RNA extraction and expressional analysis of candidate genes by qRT-PCR

Total RNA was extracted from completely washed rice seedlings treated with PEG6000 for 8 days using TRIzol Reagent (Thermo Fisher Scientific—CN). For Quantitative real-time PCR, approximately 1 µg of total RNA was reverse-transcribed by cDNA Synthesis SuperiorMix from Yeasen Biotechnology. Real time-PCR was performed in 20 µl with All-in-One™ qRT-PCR Detection Kit (Yeasen, Shanghai, China) using CFX96 Real-Time System (Bio-Rad company). The list of oligonucleotide primers used for each gene was listed in Table S1. Oligonucleotide primers for ubiquitin gene (LOC_Os03g13170) were used as the internal control for establishing equal amounts of cDNA in all reactions. The reactions were performed using the following cycle conditions, an initial 95 °C for 5 min, followed by 40 cycles of 95 °C for 10 s, 60 °C for 20 s, and 72 °C for 20 s. After obtaining the CT-value for each reaction, the relative expression was calculated by 2^-delta Ct method.

### Sequence alignment, cis-acting element prediction of promoter and homology modeling of protein

The candidate gene in 9311 and *O. longistaminata* was cloned using PCR and sequenced. With the gene in the 9311 genome or *O. longistaminata* as a reference, sequenced CDS sequence and promoter sequence of candidate gene loaded from genome database was aligned by DNAMAN software. The cis-acting regulatory element prediction of promoter sequence and homology modelling of protein were carried out by an online tool [35, 36].

### Study protocols and plant materials comply with relevant institutional, national, and international guidelines and legislation

The study protocols and plant materials used in the study comply with relevant institutional, national, and international guidelines and legislation.

## Supplementary Information

**Additional file 1. **

## Data Availability

The all datasets supporting the conclusions of this article are include in the article and supplementary information files. The whole-genome sequencing data of *O. longistaminata* was unpublished data. A total of 2,432 bin markers was used to construct the genetic linkage map covering the whole genome as described by Jin et al. [33]. The 9311 genome was used RIGW (Rice Information GateWay, http://rice.hzau.edu.cn/rice_rs1/). The gene annotation in 9311 referred to RIGW. The homology modelling of MH08g0242800 protein referred to crystal structure of ClpP1 (RCSB PDB: 7M1M, https://www.rcsb.org/structure/7M1M), which is a best reference structure model selected through online tool [36]. All raw data during the current study are available from the corresponding author on reasonable request from https://pan.baidu.com/s/1GiHFVF5GY45PUebXr56heA. Statement: the database (s) is closed, so before access to this database (s), please request the password from the corresponding author.

## Abbreviations

QTL: Quantitative trait loci
BILs: Backcross inbred lines
PCA: Principal component analysis
CV: Coefficient of variation
SD: Standard error
PV: Power vector
CR: Contribution rate
Chr: Chromosome
Exp: Experiment
PVE: Phenotypic variance explain
LOD: Logarithm of odds
CDS: Coding sequence
SNP: Single nucleotide polymorphism
