# Supplementary material for: Favorable QTLs from Oryza longistaminata improve rice drought resistance
Source: BMC Plant Biol. 2022 Mar 23;22:136. doi: 10.1186/s12870-022-03516-w (PMC8941802; doi:10.1186/s12870-022-03516-w)
Supplement: Supplementary file 1 — Additional file 1. [file 12870_2022_3516_MOESM1_ESM.docx]

**Supplementary materials**


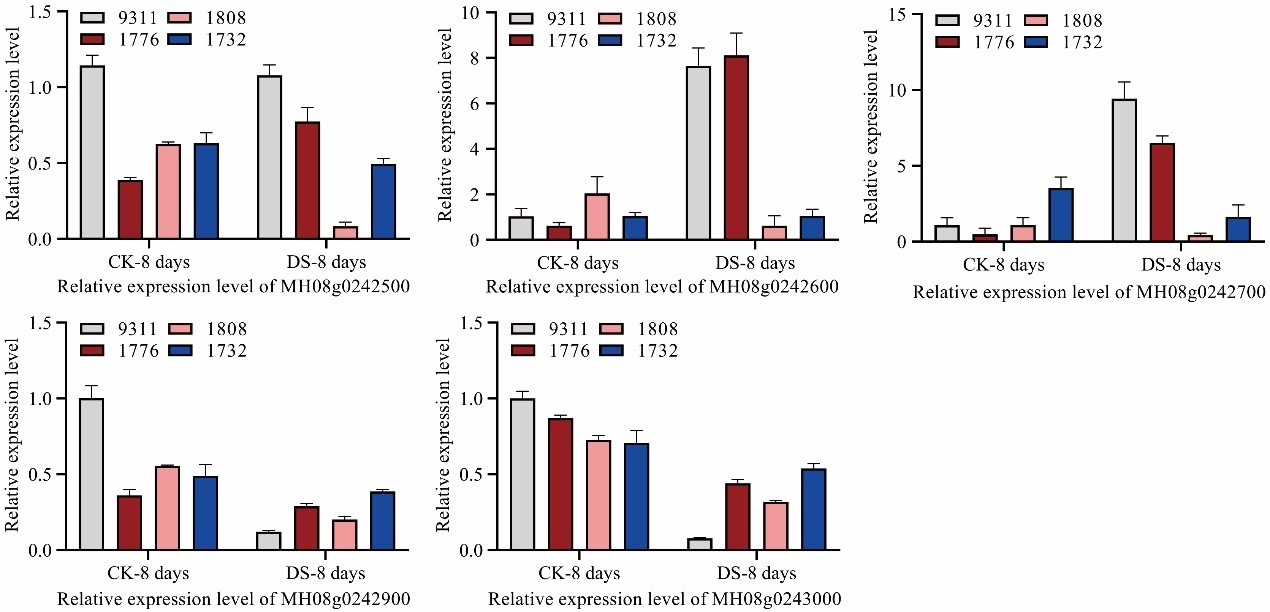


Figure S1. Relative expression level of putative genes in *qDWR8.1*. 9311 as CK, 1776 as high drought resistance of line, 1808 as low drought resistance of line, 1732 as middle drought resistance of line.


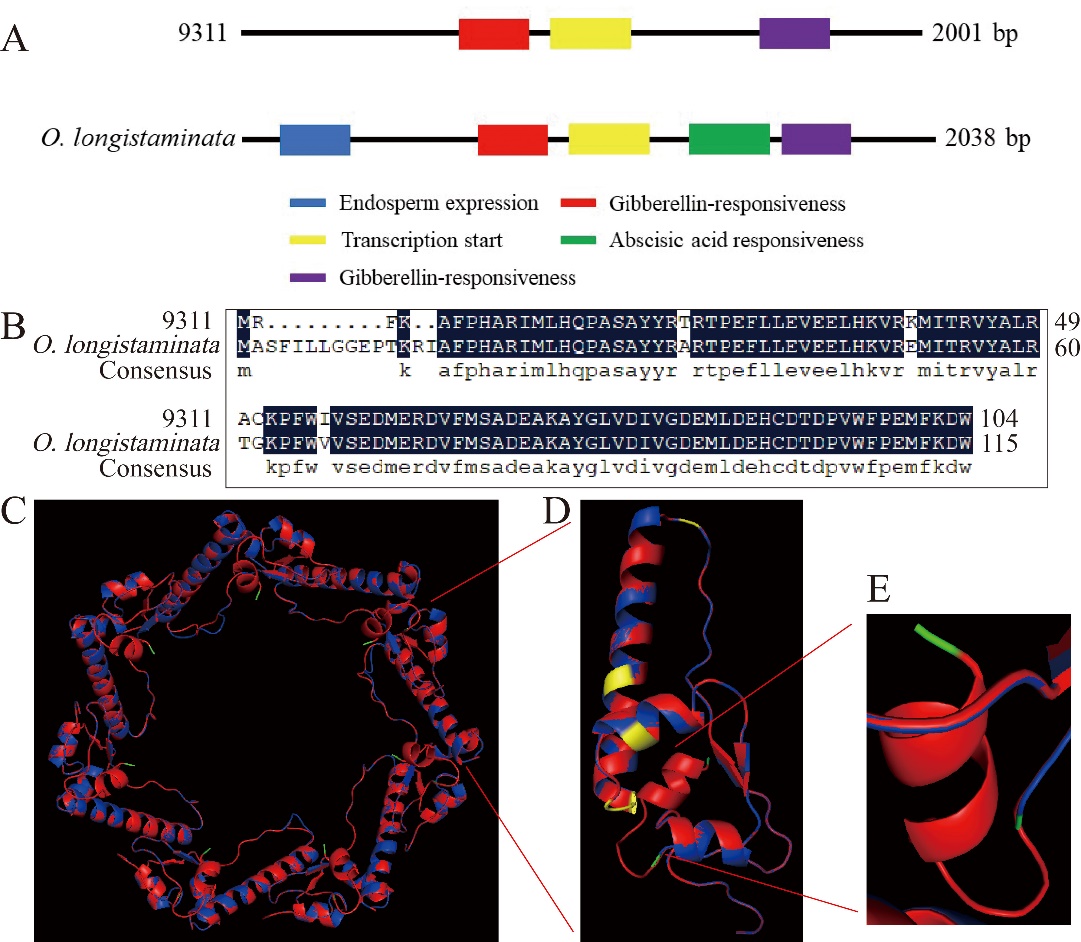


Figure S2. The MH08g0242800 promoter cis-acting elements, sequence variations and homology modeling of protein in 9311 and *O. longistaminata*. A, Predicted cis-acting elements in promoter. B, Amino acids of the MH08g0242800 encoded protein. C~E, Homology modeling of the MH08g0242800 encoded protein. Blue or red means the homology model of MH08g0242800 protein structure in 9311 or *O. longistaminata*. Yellow means the amino acid residues caused by SNP variations, Green means N terminal of protein.

Table S1. The primers of qRT-PCR to candidate genes.

| Primer name | Forward primer (5’-3’) | Reverse primer (5’-3’) | Purpose |
| --- | --- | --- | --- |
| 242500 | GCCCTCCTCCTCTCCATTTG | TCATACTGCCTTCACCGCCA | qRT-PCR |
| 242600 | ATGAAGTTCAAGATGATGGC | ACTAAGCCGATCCCAGAGAG | qRT-PCR |
| 242700 | ATCTCCGCTCGGATGAGATT | ATCACGGAAGCTGCTTAGGA | qRT-PCR |
| 242800 | TATGCACTAAGAGCATGCAA | AAACCACACTGGATCAGTAT | qRT-PCR |
| 242900 | TTGCAAACCCAAACAACCTT | TCCTTGTTCACTAATAAATCG | qRT-PCR |
| 243000 | TCAATAAACGCGTGCCAGTG | ACGCAGAGAGGAGTTCATCA | qRT-PCR |

Table S2. Damage rate of each phenotypic trait of BILs under artificial drought stress

| Exp | Trait or Damage rate (%) | Superior ratio (%) | 9311 | BILs |  |  |  |  |
| --- | --- | --- | --- | --- | --- | --- | --- | --- |
|  |  |  |  | Mean | Max. | Min. | S.D. | CV% |
| Exp.1 | LD-T | 55.24 |  |  |  |  |  |  |
|  | LR-T | 39.86 |  |  |  |  |  |  |
|  | LN | 34.26 | 13.33 | 18.79 | 40.00 | 0.00 | 5.12 | 0.27 |
|  | DWR | 30.77 | -63.81 | -52.42 | 30.97 | -162.90 | 42.44 | -0.81 |
|  | DWS | 26.57 | -2.32 | 10.82 | 45.78 | -50.43 | 18.26 | 1.69 |
|  | RL | 72.03 | 14.39 | 5.08 | 47.45 | -34.20 | 15.92 | 3.13 |
|  | SL | 28.67 | 21.08 | 24.58 | 39.33 | -7.21 | 7.13 | 0.29 |
|  | LD-T | 69.93 |  |  |  |  |  |  |
|  | LR-T | 31.47 |  |  |  |  |  |  |
| Exp.2 | LN | 53.85 | 20.00 | 15.46 | 30.77 | 0.00 | 6.62 | 0.43 |
|  | DWR | 42.66 | -53.47 | -45.84 | 24.52 | -124.69 | 28.94 | -0.63 |
|  | DWS | 46.85 | 4.44 | 4.40 | 44.68 | -53.61 | 15.96 | 3.63 |
|  | RL | 39.86 | -15.92 | -14.06 | 27.07 | -56.11 | 13.27 | -0.94 |
|  | SL | 34.27 | 19.97 | 22.48 | 41.10 | -1.33 | 7.06 | 0.31 |

Notes: LD means leaf drying, LR means leaf rolling, LN means leaf number, DWR means dry weight of root, DWS means dry weight of shoot, RL means maximum of root length, SL means maximum of shoot length, RS ratio means value between maximum root length and shoot length. CV% means the coefficient of variation. S.D. means standard error. Max. means maximum. Min. means minimum. Exp. means experiment. Superior ratio means the percentage of BILs superior to 9311 in a certain trait.

Table S3. Part of BILs with at least five agronomic traits superior to 9311 based on damage rate.

| Exp | Code | LD-T | LR-T | Damage rate (%) | | | | |
| --- | --- | --- | --- | --- | --- | --- | --- | --- |
|  |  |  |  | LN | DWR | DWS | RL | SL |
| Exp.1 | 1713 | 3.67 | 3.67 | 40 | -74.32 | 13.03 | 12.54 | 19.22 |
|  | 1726 | 3.67 | 3.00 | 13.33 | -107.23 | -7.75 | -5.62 | 33.54 |
|  | 1733 | 2.33 | 1.67 | 20 | -39.47 | -50.43 | -9.92 | 18.39 |
|  | 1744 | 3.67 | 3.67 | 20 | -80.00 | -18.91 | -17.60 | 19.66 |
|  | 1750 | 3.67 | 3.67 | 20 | -76.39 | -21.33 | -2.73 | -7.21 |
|  | 1756 | 3.00 | 3.00 | 13.16 | -96.00 | -2.64 | 2.20 | 19.40 |
|  | 1762 | 2.33 | 1.67 | 7.69 | -142.11 | -12.41 | -13.45 | 21.55 |
|  | 1763 | 3.00 | 3.00 | 23.08 | -119.23 | -13.67 | -31.18 | 21.83 |
|  | 1770 | 1.00 | 1.67 | 15.39 | -144.44 | -21.37 | -17.36 | 14.80 |
|  | 1786 | 3.67 | 5.67 | 13.33 | -137.14 | -44.24 | 1.51 | 17.18 |
|  | 1792 | 2.33 | 3.00 | 20 | -117.05 | -13.96 | -1.71 | 8.90 |
|  | 1805 | 3.00 | 3.00 | 14.29 | -148.28 | -7.65 | -18.79 | 22.71 |
|  | 1810 | 3.00 | 3.00 | 20 | -162.07 | -30.03 | -15.04 | 28.57 |
|  | 9311 | 4.33 | 5.00 | 13.33 | -63.81 | -2.32 | 14.39 | 21.08 |
| Exp.2 | 1698 | 4.33 | 4.33 | 14.29 | -61.97 | -25.99 | -6.04 | 14.14 |
|  | 1699 | 4.33 | 4.33 | 14.29 | -62.67 | 1.48 | -24.82 | 19.07 |
|  | 1702 | 5.00 | 5.00 | 7.69 | -62.38 | -0.43 | -23.18 | 21.14 |
|  | 1724 | 5.67 | 5.00 | 8.33 | -70.00 | -1.26 | 8.65 | 15.33 |
|  | 1732 | 4.33 | 4.33 | 14.29 | -124.69 | -30.67 | -11.13 | 19.82 |
|  | 1736 | 3.67 | 3.67 | 7.14 | -75.71 | 2.38 | -9.35 | 17.81 |
|  | 1737 | 3.00 | 4.33 | 0.00 | -90.16 | -6.35 | -23.69 | 17.40 |
|  | 1739 | 3.67 | 5.67 | 21.43 | -80.70 | -0.33 | -21.41 | 13.74 |
|  | 1740 | 3.67 | 5.00 | 7.69 | -81.03 | -19.38 | -7.33 | 18.60 |
|  | 1749 | 3.67 | 5.00 | 0.00 | -62.90 | -25.15 | -20.24 | 17.62 |
|  | 1762 | 3.00 | 1.67 | 0.00 | -120.00 | 0.45 | -16.68 | 17.80 |
|  | 1763 | 4.33 | 4.33 | 16.67 | -66.67 | -25.22 | -5.71 | 13.59 |
|  | 1764 | 5.00 | 7.00 | 7.14 | -83.61 | 8.66 | -33.66 | 4.63 |
|  | 1770 | 2.33 | 1.67 | 8.33 | -65.79 | -16.00 | -16.35 | 15.13 |
|  | 1771 | 3.67 | 2.33 | 20.00 | -97.78 | -11.27 | -24.27 | 17.56 |
|  | 1780 | 3.67 | 3.67 | 25 | -84.91 | -11.84 | -10.29 | 19.81 |
|  | 1781 | 3.67 | 3.67 | 0.00 | -53.62 | 3.54 | -0.74 | 18.71 |
|  | 1826 | 3.67 | 5.00 | 14.29 | -73.24 | -8.81 | -16.68 | 17.58 |
|  | 1835 | 3.00 | 3.00 | 13.33 | -70.42 | -20.33 | -41.13 | 18.60 |
|  | 1840 | 5.00 | 3.67 | 7.69 | -108.00 | -8.29 | -32.14 | 25.22 |
|  | 9311 | 5.67 | 5.00 | 20.00 | -53.47 | 4.44 | -15.92 | 19.97 |

Notes: LD means leaf drying, LR means leaf rolling, LN means leaf number, DWR means dry weight of root, DWS means dry weight of shoot, RL means maximum of root length, SL means maximum of shoot length. Exp. means experiment.

Table S4. Eigenvalues, contribution rate (CR) and cumulative contribution rate (CCR) of principal parameter resulted from 8 traits among BILs and 9311 under treatment.

| Exp. | No. | Eigenvalues | CR % | CCR % |
| --- | --- | --- | --- | --- |
| 1 | 1 | 3.27 | 40.89 | 40.89 |
|  | 2 | 1.95 | 24.43 | 65.33 |
|  | 3 | 0.98 | 12.30 | 77.63 |
| 2 | 1 | 3.33 | 41.59 | 41.59 |
|  | 2 | 1.94 | 24.30 | 65.89 |
|  | 3 | 1.20 | 14.96 | 80.84 |

CR, contribution rate; CCR, cumulative contribution rate.

Table S5. The factor scores of PCA, F value of comprehensive evaluate included CK (9311) and representative BILs lines.

| Code in Exp.1 | F1 | F2 | F3 | F value | Code in Exp.2 | F1 | F2 | F3 | F value | Rank |
| --- | --- | --- | --- | --- | --- | --- | --- | --- | --- | --- |
| 1788 | 4.00 | 0.49 | 2.16 | 2.60 | 1699 | 6.28 | -0.37 | 0.69 | 3.25 | 1 |
| 1701 | 3.58 | 0.64 | 2.06 | 2.41 | 1728 | 4.32 | 0.11 | 1.90 | 2.61 | 2 |
| 1776 | 2.48 | 2.67 | 0.72 | 2.26 | 1738 | 4.50 | -0.33 | 1.01 | 2.40 | 3 |
| 1827 | 3.61 | 0.01 | 1.30 | 2.11 | 1776 | 1.64 | 3.87 | -0.23 | 1.96 | 4 |
| 1738 | 3.99 | -0.73 | 0.55 | 1.96 | 1729 | 3.31 | 1.38 | -1.08 | 1.92 | 5 |
| 1729 | 3.36 | 1.08 | -1.02 | 1.95 | 1742 | 4.39 | -1.29 | -0.89 | 1.71 | 6 |
| 1728 | 3.82 | -0.70 | 0.61 | 1.89 | 1825 | 3.19 | 0.33 | -0.22 | 1.70 | 7 |
| 1704 | 3.69 | -0.22 | -0.72 | 1.76 | 1791 | 3.83 | -0.26 | -1.09 | 1.69 | 8 |
| 1757 | 3.75 | -0.82 | -0.05 | 1.71 | 1827 | 3.54 | -0.76 | 0.49 | 1.68 | 9 |
| 1792 | 2.75 | 0.83 | -0.84 | 1.58 | 1704 | 2.90 | 0.25 | 0.41 | 1.65 | 10 |
| 1716 | 2.96 | -0.27 | 0.34 | 1.53 | 1754 | 3.23 | 0.01 | -0.16 | 1.64 | 11 |
| 1812 | 1.59 | 1.62 | 0.83 | 1.48 | 1815 | 2.92 | 0.55 | -0.65 | 1.55 | 12 |
| 1699 | 2.90 | -0.17 | -0.24 | 1.44 | 1788 | 3.01 | -0.62 | 0.83 | 1.51 | 13 |
| 1834 | 3.07 | -0.77 | 0.28 | 1.42 | 1707 | 3.44 | -1.01 | 0.26 | 1.51 | 14 |
| 1707 | 3.10 | -0.81 | 0.11 | 1.40 | 1702 | 3.03 | -0.48 | 0.41 | 1.49 | 15 |
| 1814 | 1.58 | 1.49 | 0.34 | 1.35 | 1841 | 2.11 | 1.04 | 0.23 | 1.44 | 16 |
| 1732 | 1.46 | 1.59 | 0.41 | 1.33 | 1834 | 2.85 | -0.88 | 0.98 | 1.38 | 17 |
| 1709 | 3.29 | -1.27 | -0.38 | 1.27 | 1708 | 2.51 | 0.32 | -0.45 | 1.31 | 18 |
| 1791 | 2.30 | -0.52 | 1.26 | 1.25 | 1714 | 0.78 | 2.72 | 0.36 | 1.28 | 19 |
| 1752 | 2.70 | -0.05 | -1.02 | 1.24 | 1709 | 3.64 | -1.82 | -0.56 | 1.22 | 20 |
| 1779 | 2.90 | -1.02 | 0.15 | 1.23 | 1705 | 2.74 | -1.27 | 1.04 | 1.22 | 21 |
| 1725 | 2.75 | -0.45 | -0.57 | 1.22 | 1809 | 0.88 | 1.32 | 1.85 | 1.19 | 22 |
| 1832 | 1.77 | 0.03 | 1.52 | 1.18 | 1743 | 0.78 | 1.96 | 1.09 | 1.19 | 23 |
| 1702 | 2.54 | -0.32 | -0.39 | 1.17 | 1835 | 0.63 | 2.40 | 0.44 | 1.13 | 24 |
| 1754 | 3.13 | -0.98 | -1.26 | 1.14 | 1725 | 2.68 | -0.59 | -0.64 | 1.08 | 25 |
| 1737 | 1.17 | 1.72 | -0.23 | 1.12 | 1752 | 1.48 | 1.37 | -0.77 | 1.03 | 26 |
| 1825 | 3.22 | -1.42 | -0.99 | 1.09 | 1713 | -0.79 | 3.64 | 1.84 | 1.03 | 27 |
| 1708 | 2.49 | -0.21 | -1.35 | 1.03 | 1732 | 1.19 | 0.87 | 0.53 | 0.97 | 28 |
| 1804 | 0.31 | 2.54 | 0.42 | 1.03 | 1833 | 2.33 | -0.26 | -0.81 | 0.97 | 29 |
| 1774 | 1.32 | 1.14 | -0.28 | 1.01 | 1753 | 0.67 | 2.31 | -0.44 | 0.96 | 30 |
| 1748 | 1.63 | 0.46 | -0.25 | 0.96 | 1828 | 0.99 | 1.40 | 0.08 | 0.95 | 31 |
| 1742 | 3.04 | -2.15 | 0.13 | 0.94 | 1736 | 0.03 | 2.21 | 1.41 | 0.94 | 32 |
| 1705 | 2.52 | -1.52 | 0.02 | 0.85 | 9311 | 1.19 | 0.11 | 1.26 | 0.88 | 33 |
| 1736 | 0.47 | 2.15 | -0.51 | 0.84 | 1748 | 0.97 | 1.33 | -0.17 | 0.87 | 34 |
| 9311 | 0.21 | 1.09 | 1.73 | 0.73 | 1737 | 1.95 | 0.50 | -1.66 | 0.84 | 35 |
| 1789 | 1.66 | -1.08 | 1.07 | 0.70 | 1774 | 0.70 | 1.08 | 0.80 | 0.83 | 36 |
| 1726 | -0.12 | 1.95 | 0.88 | 0.69 | 1804 | 0.38 | 1.92 | 0.16 | 0.80 | 37 |
| 1753 | 0.60 | 1.77 | -1.32 | 0.66 | 1779 | 1.76 | -0.58 | 0.36 | 0.80 | 38 |
| 1833 | 1.28 | 0.38 | -0.92 | 0.65 | 1792 | 1.39 | 0.85 | -0.99 | 0.79 | 39 |
| 1815 | 1.72 | -0.97 | -0.33 | 0.55 | 1772 | 0.37 | 1.37 | 0.52 | 0.70 | 40 |
| 1723 | 1.81 | -0.27 | -2.37 | 0.49 | 1838 | 1.44 | -0.17 | -0.20 | 0.65 | 41 |
| 1811 | 0.31 | 0.65 | 0.78 | 0.49 | 1819 | 0.56 | 0.74 | 0.63 | 0.63 | 42 |
| 1777 | 1.64 | -1.48 | 0.55 | 0.49 | 1723 | 0.38 | 2.03 | -1.01 | 0.62 | 43 |
| 1717 | -0.20 | 1.58 | 0.39 | 0.45 | 1777 | 1.79 | -0.96 | -0.34 | 0.57 | 44 |
| 1733 | 0.24 | 1.68 | -1.31 | 0.45 | 1716 | 1.56 | -1.83 | 1.70 | 0.57 | 45 |
| 1764 | -0.41 | 1.48 | 1.11 | 0.43 | 1758 | 0.28 | 2.21 | -1.45 | 0.54 | 46 |
| 1794 | -0.69 | 2.26 | 0.33 | 0.40 | 1733 | 0.21 | 1.85 | -0.75 | 0.53 | 47 |
| 1786 | 0.51 | 0.12 | 0.55 | 0.39 | 1842 | 0.66 | 0.09 | 0.86 | 0.52 | 48 |
| 1714 | 0.16 | 1.46 | -1.13 | 0.37 | 1814 | 0.06 | 1.09 | 0.65 | 0.48 | 49 |
| 1743 | 0.11 | 1.11 | -0.25 | 0.37 | 1789 | 2.18 | -1.79 | -0.89 | 0.42 | 50 |
| 1772 | 0.47 | 0.71 | -0.67 | 0.37 | 1747 | 0.66 | 0.26 | -0.14 | 0.39 | 51 |
| 1770 | -1.02 | 3.37 | -1.24 | 0.32 | 1831 | 0.48 | 0.79 | -0.59 | 0.38 | 52 |
| 1842 | 0.35 | 0.78 | -0.73 | 0.31 | 1818 | 0.49 | 0.61 | -1.09 | 0.23 | 53 |
| 1762 | -0.46 | 2.23 | -0.99 | 0.30 | 1807 | -0.34 | 0.82 | 0.69 | 0.20 | 54 |
| 1828 | 0.43 | -0.16 | 0.78 | 0.30 | 1799 | 1.32 | -1.22 | -0.82 | 0.16 | 55 |
| 1751 | -0.42 | 1.77 | -0.27 | 0.30 | 1796 | 0.17 | 0.09 | 0.19 | 0.15 | 56 |
| 1805 | -0.66 | 2.02 | -0.03 | 0.28 | 1751 | -0.68 | 1.79 | -0.25 | 0.14 | 57 |
| 1835 | -0.52 | 2.04 | -0.70 | 0.26 | 1706 | -1.31 | -0.04 | 4.47 | 0.14 | 58 |
| 1739 | 0.45 | 0.22 | -0.35 | 0.25 | 1826 | 0.16 | 0.44 | -0.64 | 0.10 | 59 |
| 1778 | -0.16 | 0.64 | 0.65 | 0.22 | 1762 | -1.22 | 2.79 | -0.63 | 0.10 | 60 |
| 1700 | 0.31 | 0.83 | -1.29 | 0.22 | 1700 | -0.36 | 0.82 | 0.07 | 0.07 | 61 |
| 1787 | -1.39 | 2.73 | 0.38 | 0.19 | 1781 | -1.18 | 1.88 | 0.50 | 0.05 | 62 |
| 1810 | -0.42 | 1.54 | -0.58 | 0.17 | 1757 | 0.72 | -0.89 | -0.40 | 0.03 | 63 |
| 1698 | -1.33 | 2.45 | 0.59 | 0.16 | 1812 | 0.05 | 0.39 | -0.68 | 0.02 | 64 |
| 1818 | 0.70 | -0.77 | 0.05 | 0.14 | 1735 | -0.38 | 0.17 | 0.91 | 0.02 | 65 |
| 1806 | -0.93 | 1.64 | -0.05 | 0.02 | 1816 | -1.06 | 0.94 | 1.51 | 0.02 | 66 |
| 1823 | -1.34 | 0.52 | 3.49 | 0.01 | 1839 | 0.79 | -1.81 | 0.79 | 0.01 | 67 |
| 1807 | -0.08 | 0.53 | -0.77 | 0.00 | 1715 | -0.65 | 0.15 | 1.54 | 0.00 | 68 |
| 1750 | -0.34 | 0.75 | -0.34 | 0.00 | 1722 | 0.53 | -0.46 | -1.04 | -0.06 | 69 |
| 1735 | -0.28 | -0.02 | 0.85 | -0.01 | 1749 | -1.75 | 1.82 | 1.57 | -0.06 | 70 |
| 1841 | 0.37 | 0.05 | -1.47 | -0.02 | 1830 | -1.37 | 0.83 | 1.91 | -0.10 | 71 |
| 1816 | -0.47 | 1.03 | -0.63 | -0.03 | 1771 | -0.16 | 1.17 | -2.07 | -0.11 | 72 |
| 1765 | -0.75 | 0.64 | 1.02 | -0.03 | 1703 | -1.30 | 0.68 | 1.66 | -0.16 | 73 |
| 1838 | 0.53 | -1.45 | 0.66 | -0.07 | 1701 | -0.27 | -0.24 | 0.12 | -0.19 | 74 |
| 1781 | -1.13 | 1.45 | 0.29 | -0.09 | 1739 | 0.67 | -0.63 | -2.00 | -0.21 | 75 |
| 1720 | 0.37 | -0.98 | 0.06 | -0.10 | 1726 | -0.03 | 0.13 | -1.26 | -0.21 | 76 |
| 1819 | 0.09 | -1.02 | 1.01 | -0.11 | 1820 | -0.86 | -0.57 | 2.16 | -0.21 | 77 |
| 1744 | -1.10 | 1.37 | 0.13 | -0.13 | 1764 | -0.04 | -0.86 | 0.29 | -0.23 | 78 |
| 1799 | 0.95 | -2.12 | 0.24 | -0.13 | 1734 | -0.35 | -0.65 | 0.73 | -0.24 | 79 |
| 1797 | -1.19 | 1.10 | 0.92 | -0.13 | 1832 | 0.33 | -0.96 | -0.71 | -0.25 | 80 |
| 1763 | -1.94 | 2.80 | -0.24 | -0.18 | 1698 | 0.09 | -0.12 | -1.54 | -0.27 | 81 |
| 1734 | -0.47 | 0.46 | -0.53 | -0.19 | 1745 | -0.80 | 1.08 | -1.03 | -0.28 | 82 |
| 1773 | -0.95 | 0.80 | -0.10 | -0.27 | 1760 | -0.96 | 0.22 | 0.72 | -0.29 | 83 |
| 1715 | -0.44 | -0.77 | 1.29 | -0.27 | 1840 | -1.43 | 1.13 | 0.53 | -0.30 | 84 |
| 1756 | -1.65 | 2.19 | -0.61 | -0.27 | 1717 | -0.41 | -0.30 | 0.02 | -0.30 | 85 |
| 1745 | -0.92 | 1.32 | -1.34 | -0.28 | 1740 | -1.73 | 1.38 | 0.76 | -0.34 | 86 |
| 1783 | -0.93 | 0.16 | 0.88 | -0.30 | 1712 | -0.83 | -1.07 | 2.15 | -0.35 | 87 |
| 1758 | -0.99 | 1.73 | -2.09 | -0.31 | 1810 | -0.71 | 1.47 | -2.32 | -0.35 | 88 |
| 1760 | -0.64 | 0.67 | -1.26 | -0.33 | 1803 | -0.25 | -0.10 | -1.20 | -0.38 | 89 |
| 1840 | -1.18 | 1.07 | -0.29 | -0.33 | 1836 | -1.29 | 0.74 | 0.31 | -0.38 | 90 |
| 1795 | -0.19 | -1.26 | 0.83 | -0.37 | 1741 | -0.50 | -0.56 | 0.15 | -0.40 | 91 |
| 1784 | -0.11 | -1.15 | 0.21 | -0.38 | 1770 | -2.09 | 2.99 | -1.22 | -0.40 | 92 |
| 1839 | 0.05 | -1.41 | 0.04 | -0.41 | 1765 | -0.64 | -1.08 | 1.21 | -0.43 | 93 |
| 1741 | -0.62 | -0.06 | -0.42 | -0.42 | 1811 | 0.35 | -1.58 | -0.83 | -0.45 | 94 |
| 1740 | -0.65 | -0.50 | 0.52 | -0.42 | 1794 | -1.31 | 0.36 | 0.34 | -0.50 | 95 |
| 1747 | -0.36 | -0.42 | -0.95 | -0.47 | 1805 | -1.03 | 0.72 | -1.03 | -0.51 | 96 |
| 1836 | -1.50 | 0.36 | 1.15 | -0.49 | 1773 | -0.61 | -0.46 | -0.33 | -0.51 | 97 |
| 1801 | -1.03 | -0.44 | 1.05 | -0.51 | 1778 | -1.59 | 0.54 | 0.47 | -0.57 | 98 |
| 1820 | -0.76 | -0.64 | 0.53 | -0.52 | 1783 | -1.60 | 0.82 | -0.22 | -0.62 | 99 |
| 1771 | -1.20 | 0.23 | -0.20 | -0.59 | 1837 | -0.86 | -0.99 | 0.55 | -0.64 | 100 |
| 1829 | -2.41 | 1.31 | 1.63 | -0.60 | 1719 | -2.27 | 1.78 | -0.15 | -0.66 | 101 |
| 1706 | -1.16 | -0.50 | 0.83 | -0.64 | 1711 | -1.00 | -2.37 | 2.92 | -0.68 | 102 |
| 1775 | -2.47 | 1.05 | 1.97 | -0.66 | 1806 | -1.31 | 0.49 | -0.91 | -0.70 | 103 |
| 1718 | 0.29 | -2.79 | 0.43 | -0.66 | 1775 | -1.68 | -0.08 | 0.82 | -0.73 | 104 |
| 1826 | -0.44 | -1.08 | -0.57 | -0.66 | 1756 | -2.32 | 1.43 | 0.05 | -0.75 | 105 |
| 1780 | -2.07 | 1.39 | -0.10 | -0.67 | 1724 | -0.49 | -0.93 | -1.26 | -0.77 | 106 |
| 1790 | -1.15 | -0.54 | 0.61 | -0.68 | 1829 | -1.86 | 0.11 | 0.48 | -0.83 | 107 |
| 1746 | -0.57 | -1.22 | -0.56 | -0.78 | 1755 | -0.21 | -1.43 | -1.65 | -0.84 | 108 |
| 1749 | -1.95 | 0.53 | 0.52 | -0.78 | 1780 | -2.72 | 1.81 | 0.01 | -0.85 | 109 |
| 1731 | -1.95 | 0.34 | 0.82 | -0.79 | 1730 | -0.35 | -1.80 | -0.78 | -0.87 | 110 |
| 1759 | -1.25 | 0.35 | -1.74 | -0.82 | 1821 | -1.62 | -0.36 | 0.28 | -0.89 | 111 |
| 1796 | -1.98 | 0.06 | 1.25 | -0.82 | 1787 | -2.72 | 1.35 | 0.50 | -0.90 | 112 |
| 1766 | -1.85 | 0.46 | 0.04 | -0.83 | 1782 | -1.10 | -2.24 | 1.81 | -0.91 | 113 |
| 1769 | -0.85 | -1.88 | 1.21 | -0.85 | 1769 | -1.42 | -1.35 | 1.15 | -0.92 | 114 |
| 1803 | -0.91 | -0.59 | -1.22 | -0.86 | 1795 | -0.87 | -1.51 | -0.35 | -0.97 | 115 |
| 1727 | -1.87 | -0.69 | 2.11 | -0.87 | 1718 | 0.04 | -3.07 | -0.38 | -0.97 | 116 |
| 1713 | -1.68 | 0.91 | -1.87 | -0.89 | 1823 | -1.41 | -1.85 | 1.67 | -0.97 | 117 |
| 1785 | -1.55 | -0.13 | -0.47 | -0.93 | 1801 | -1.86 | -0.33 | 0.42 | -0.98 | 118 |
| 1722 | 0.07 | -2.65 | -0.84 | -0.93 | 1790 | -0.98 | -1.47 | -0.19 | -0.98 | 119 |
| 1782 | -1.05 | -1.97 | 1.51 | -0.93 | 1721 | -0.70 | -1.07 | -1.70 | -1.00 | 120 |
| 1712 | -1.41 | -0.97 | 0.66 | -0.94 | 1750 | -1.99 | 1.19 | -1.88 | -1.02 | 121 |
| 1730 | -1.07 | -0.87 | -0.85 | -0.97 | 1797 | -1.95 | -0.35 | 0.34 | -1.04 | 122 |
| 1724 | -0.30 | -2.52 | -0.29 | -1.00 | 1817 | -0.85 | -1.77 | -0.75 | -1.11 | 123 |
| 1761 | -1.86 | -0.10 | 0.03 | -1.01 | 1759 | -1.48 | 0.01 | -1.95 | -1.12 | 124 |
| 1830 | -2.09 | -0.59 | 1.70 | -1.02 | 1813 | -1.63 | -1.02 | -0.23 | -1.19 | 125 |
| 1767 | -0.32 | -2.46 | -1.02 | -1.10 | 1784 | -0.95 | -2.58 | 0.34 | -1.20 | 126 |
| 1809 | -1.94 | -0.31 | -0.20 | -1.15 | 1766 | -1.49 | -2.02 | 0.70 | -1.25 | 127 |
| 1703 | -1.42 | -0.55 | -1.71 | -1.19 | 1744 | -2.24 | 0.43 | -1.22 | -1.25 | 128 |
| 1711 | -1.13 | -2.34 | 0.67 | -1.23 | 1720 | -1.41 | -0.51 | -2.15 | -1.28 | 129 |
| 1793 | -2.02 | -0.64 | -0.38 | -1.32 | 1763 | -3.28 | 1.23 | 0.11 | -1.30 | 130 |
| 1831 | -1.48 | -1.62 | -0.39 | -1.35 | 1822 | -2.02 | -0.38 | -1.18 | -1.37 | 131 |
| 1802 | -1.51 | -2.32 | 0.69 | -1.41 | 1800 | -1.37 | -2.35 | 0.05 | -1.40 | 132 |
| 1800 | -1.69 | -1.37 | -0.68 | -1.43 | 1710 | -1.64 | -2.02 | -0.07 | -1.46 | 133 |
| 1721 | -0.51 | -3.39 | -0.99 | -1.49 | 1767 | -1.08 | -2.27 | -1.22 | -1.47 | 134 |
| 1821 | -2.14 | -1.30 | -0.08 | -1.55 | 1761 | -2.37 | -1.21 | 0.56 | -1.48 | 135 |
| 1813 | -3.10 | -0.47 | 1.18 | -1.60 | 1746 | -1.51 | -0.98 | -2.47 | -1.53 | 136 |
| 1719 | -2.62 | -0.27 | -0.90 | -1.61 | 1786 | -2.51 | -1.01 | 0.29 | -1.54 | 137 |
| 1755 | -1.28 | -2.76 | -1.54 | -1.79 | 1731 | -1.64 | -1.92 | -0.68 | -1.55 | 138 |
| 1768 | -2.43 | -1.60 | -0.72 | -1.90 | 1793 | -1.86 | -1.78 | -0.39 | -1.56 | 139 |
| 1822 | -2.98 | -1.60 | 0.80 | -1.95 | 1727 | -1.83 | -2.43 | 0.37 | -1.60 | 140 |
| 1710 | -2.33 | -1.40 | -1.82 | -1.96 | 1802 | -1.17 | -3.18 | -0.81 | -1.71 | 141 |
| 1817 | -2.92 | -2.92 | 0.83 | -2.33 | 1768 | -1.68 | -2.60 | -0.58 | -1.75 | 142 |
| 1837 | -3.02 | -2.63 | -2.10 | -2.75 | 1785 | -3.59 | 0.27 | -0.34 | -1.83 | 143 |
| 1808 | -4.25 | -2.21 | -0.79 | -3.06 | 1808 | -3.36 | -1.28 | -1.13 | -2.32 | 144 |

Notes: F1, F2 and F3 represent factor 1, factor 2 and factor 3. Exp. means experiment.

Table S6. QTLs for drought resistance from 9311 with negative effect under artificial stress.

| Trait | QTLs | Exp. | Chr. | Position | L-Marker | R-Marker | LOD | PVE (%) | Add |
| --- | --- | --- | --- | --- | --- | --- | --- | --- | --- |
| LR | *qLR9.1* | 2 | 9 | 20.47 (cM) | BIN9-22 | BIN9-23 | 2.76 | 7.39 | -0.99 |
| LN | *qLN7.1* | 2 | 7 | 123.5 (cM) | BIN7-84 | BIN7-85 | 4.46 | 3.84 | -0.08 |
|  | *qLN8.1* | 2 | 8 | 99.58 (cM) | BIN8-80 | BIN8-81 | 2.92 | 4.82 | -0.31 |
|  | *qLN8.2* | 2 | 8 | 113.58 (cM) | BIN8-95 | BIN8-96 | 22.63 | 28.59 | -0.33 |
|  | *qLN9.1* | 1 | 9 | 18.47 (cM) | BIN9-16 | BIN9-17 | 2.86 | 6.33 | -0.18 |
|  | *qLN9.2* | 2 | 9 | 72.47 (cM) | BIN9-53 | BIN9-54 | 2.88 | 4.93 | -0.44 |
| DWR | *qDWR2.1* | 2 | 2 | 47.43 (cM) | BIN2-51 | BIN2-52 | 4.28 | 0.74 | -1.04 |
|  | *qDWR5.1* | 2 | 5 | 198.97 (cM) | BIN5-156 | BIN5-157 | 50.32 | 22.18 | -6.90 |
|  | *qDWR6.1* | 1 | 6 | 113.93 (cM) | BIN6-89 | BIN6-90 | 3.51 | 5.40 | -2.78 |
|  | *qDWR7.1* | 2 | 7 | 110.5 (cM) | BIN7-80 | BIN7-81 | 2.98 | 0.50 | -0.94 |
| RL | *qRL2.1* | 1 | 2 | 283.43 (cM) | BIN2-273 | BIN2-274 | 2.92 | 7.43 | -0.56 |
|  | *qRL7.1* | 2 | 7 | 110.5 (cM) | BIN7-80 | BIN7-81 | 2.96 | 9.14 | -0.58 |
|  | *qRL9.1* | 1 | 9 | 161.47 (cM) | BIN9-104 | BIN9-105 | 2.59 | 6.24 | -1.57 |
| Rs ratio | *qRSratio1.1* | 1 | 1 | 212.45 (cM) | BIN1-152 | BIN1-153 | 19.31 | 43.60 | -0.07 |
|  | *qRSratio1.2* | 2 | 1 | 213.45 (cM) | BIN1-154 | BIN1-155 | 16.69 | 42.13 | -0.08 |

Note: PVE means percentage of phenotypic variance explained by each QTL. LOD means logarithm of odds. Exp. means experiment. Chr. means chromosome.

Table S7. The rank and genotypic analysis of repeated six BILs in the two experiments based on comprehensive evaluate and QTLs analysis.

| Code | Rank in Exp.1 | Rank in Exp.2 | QTLs from *O. longistaminata* | QTLs from 9311 |
| --- | --- | --- | --- | --- |
| 1702 | 24 | 15 | *qDWR1.1, qLD7.2,*  *qDWS8.1, qDWR8.1* | *qRL2.1, qDWR5.1, qDWR6.1, qDWR7.1,*  *qRL7.1, qLN7.1, qLN8.1, qLN8.2, qLN9.1,*  *qLR9.1, qLN9.2, qRL9.1* |
| 1704 | 8 | 10 | *qDWR1.1, qDWS1.1,*  *qDWS8.1, qDWR8.1* | *qDWR2.1, qRL2.1, qDWR6.1, qDWR7.1,*  *qRL7.1, qLN7.1, qLN8.1, qLN8.2, qLN9.1,*  *qLR9.1, qLN9.2, qRL9.1* |
| 1728 | 7 | 2 | *qDWR1.1,*  *qDWS1.1, qDWR4.1,*  *qDWS8.1, qDWR8.1* | *qDWR2.1, qRL2.1, qDWR5.1, qDWR6.1,*  *qDWR7.1, qRL7.1, qLN7.1, qLN8.2, qLN9.1,*  *qLR9.1, qLN9.2, qRL9.1* |
| 1732 | 17 | 28 | *qDWS1.1,*  *qDWS8.1, qDWR8.1* | *qRSratio1.1, qRSratio1.2, qDWR2.1, qRL2.1, qDWR5.1, qDWR6.1, qDWR7.1, qRL7.1, qLN7.1, qLN8.1, qLN8.2, qLN9.1, qLR9.1, qLN9.2, qRL9.1* |
| 1742 | 32 | 6 | *qDWR1.1, qDWS1.1,*  *qDWS8.1, qDWR8.1* | *qDWR2.1, qRL2.1, qDWR5.1, qDWR6.1,*  *qDWR7.1, qRL7.1, qLN7.1, qLN8.1, qLN8.2,*  *qLN9.1, qLR9.1, qLN9.2, qRL9.1* |
| 1776 | 3 | 4 | *qDWS8.1, qDWR8.1* | *qRSratio1.1, qRSratio1.2, qDWR2.1, qRL2.1, qDWR5.1, qDWR6.1, qDWR7.1, qRL7.1, qLN7.1, qLN8.1, qLN8.2, qLN9.1, qLR9.1, qLN9.2, qRL9.1* |
| 9311 | 35 | 33 |  |  |
